# Advancing Conservative Treatment of Knee Osteoarthritis: 3D‐Printed Shoe Soles for Passive Toe‐Out Gait Modification

**DOI:** 10.1002/jfa2.70174

**Published:** 2026-06-22

**Authors:** Ziang Jiang, Paciane Bo Studer, Matthias Zäh, Christian Kryenbühl, Stephan Reichenbach, William R. Taylor, Qiang Zhang

**Affiliations:** ^1^ Institute for Biomechanics ETH Zürich Zürich Switzerland; ^2^ Swissbiomechanics AG Einsiedeln Switzerland; ^3^ Institute of Social and Preventive Medicine University of Bern Bern Switzerland; ^4^ Department of Rheumatology and Immunology Inselspital Bern University Hospital University of Bern Bern Switzerland

**Keywords:** 3D‐printed shoe soles, foot orthosis, gait modification, joint kinetics, knee osteoarthritis

## Abstract

**Introduction:**

To evaluate whether a 3D‐printed shoe sole can passively replicate toe‐out gait biomechanics and reduce the knee adduction moment (KAM) and knee adduction angular impulse (KAAI), addressing limitations of traditional gait retraining.

**Methods:**

Custom shoe soles were 3D‐printed using gyroid infill structures to create a rotational hind‐sole and a variable stiffness fore‐sole (‘RVS’ shoes). Twenty‐one healthy adults completed three baseline gait trials in control shoes, followed by randomised biofeedback‐based gait retraining at toe‐out angles of 5°, 10° and 15°. After each retraining session, motion capture was used during overground walking. Participants then walked wearing RVS shoes for three trials. Gait biomechanics were analysed using repeated measures ANOVA and multiple linear regression.

**Results:**

Compared to baseline, KAM's second peak was significantly reduced after toe‐out retraining: 5° (13.6%, *p* = 0.002), 10° (20.2%, *p* < 0.001) and 15° (31.7%, *p* < 0.001). RVS shoes alone reduced it by 16.3% (*p* < 0.001), and KAAI by 8.6% (*p* < 0.001)—a reduction not achieved by retraining. Regression analysis showed that changes in KAM were strongly predicted (95%) by knee centre‐of‐pressure offset and mediolateral ground reaction force.

**Conclusion:**

3D‐printed RVS shoes passively replicated or exceeded the biomechanical effects of active toe‐out gait retraining, offering a promising, low‐effort intervention for managing knee osteoarthritis.

## 1 Introduction

Knee osteoarthritis (KOA) is a highly prevalent degenerative joint disease [1, 2], affecting over 26% of individuals over 45 years [1]. Increased knee loading has been identified as a key aetiological factor in KOA progression [3, 4], particularly in respect to excessive compressive forces that accelerate cartilage degeneration and subchondral bone deterioration [5]. Here, the knee adduction moment (KAM) serves as a widely accepted surrogate measure of medial knee loading [6] and has been demonstrated to strongly correlate with KOA severity, cumulative loading, and cartilage loss [7].

Biomechanical treatments have become core modalities for the conservative management of KOA, and are endorsed by prominent healthcare organisations, including the National Institute for Health and Care Excellence [8], and the Osteoarthritis Research Society International [9]. One such approach, the gait modification intervention, involves modifying foot progression angle (FPA) towards deliberate toe‐in/out gait patterns [10] that can shift the foot COP and shorten the frontal‐plane ground reaction force (GRF) vector around the knee joint centre [11, 12]. In general, toe‐in gait predominantly affects early‐stance, while toe‐out effectively reduces the late‐stance peak KAM [13, 14]. While only little evidence has been reported on the efficacy of toe‐in gait patterns for reducing pain in KOA [15], studies employing 7.5°–16.6° toe‐out gait modifications have demonstrated reductions in the late‐stance peak KAM of up to 37.5% [12, 13], and shown clinical feasibility with successful retraining and adherence over time [16, 17].

Recent studies have identified a strong correlation between the late‐stance peak KAM and increased medial meniscus extrusion in KOA [18], which has been successfully inhibited by toe‐out gait modification [19]. Given the critical role of the menisci in mediating the stress‐loads [20] and the consequences of meniscus extrusion in progressing knee OA [21], the potential for toe‐out gait modifications to retard the progression of KOA is clear.

Despite its documented effectiveness, gait retraining is limited by poor adherence, as patients often fail to maintain the prescribed modification in real‐world settings. Effective training in FPA modification is reliant on continuous tracking and real‐time feedback for adjustment adherence [22, 23]. Even with full compliance, however, at least 6‐week adherence is typically required for apparent and stable effects [22, 23, 24], and clinical benefits derived from gait retraining are generally not sustained after training cessation [25]. As an alternative, smart devices integrated with vibratory inertial measurement units have been recently introduced to track FPA and provide vibrotactile feedback to alert users of deviations from the target FPA during gait [24, 26]. Nevertheless, potential limitations of such technologies include their high cost [27] and lack of validation in free living environments [22, 28].

One plausible solution is to fabricate an orthotic shoe capable of passively inducing FPA modification through implementing a ‘rotational unit’ beneath the shoe sole. 3D printing offers high structural and morphological freedom, and may be suitable to achieve such a distinctive shoe sole geometry as well as integrate variable stiffness properties. Therefore, the objective of this study was to develop a 3D‐printed shoe sole combined with rotational and variable stiffness modules (the so‐called RVS shoe sole), capable of inducing toe‐out modifications during ambulation, towards modifying lower‐limb joint kinematics and kinetics and reducing the late‐stance peak KAM. We hypothesised:

1. Comparable with toe‐out gait retraining, the RVS shoes are capable of modifying the subjects' FPA towards increased toe‐out even without real‐time feedback as well as reducing the late‐stance peak KAM and knee adduction angular impulse (KAAI).
2. Modifications to the COP and mediolateral GRF are the main contributors to any changes in the late‐stance peak KAM.

## 2 Material and Methods

### 2.1 Participants

The study sample size was estimated using G*Power software (Version 3.1.3). An effect size of 0.3, estimated from the biomechanical effect of walking with different FPAs [29], was applied to a power analysis for repeated measures of analysis of variance (RM‐ANOVA). This analysis indicated that a minimum of 19 subjects would be required to achieve a statistical power of 90% (α level of 0.05) to detect biomechanical differences. Accordingly, 21 healthy individuals (12 females, 9 males; aged: 26.8 ± 6.5 years; height: 1.73 ± 0.07 m; body mass: 69.7 ± 12.2 kg) were recruited, excluding those with foot/ankle injury within the previous 6 months and/or previous lower‐limb surgery. This study was conducted in accordance with the Declaration of Helsinki. Ethical approval was obtained from the institutional Ethics Committee, and all subjects provided written informed consent.

### 2.2 Design and Manufacture of Shoe Sole

**FIGURE 1:**
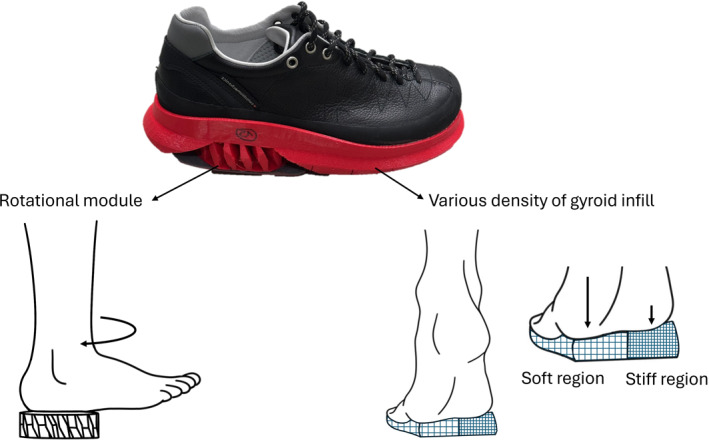
3D‐printed shoes with region‐specific geometric and stiffness characteristics. A rotation module was implemented for the hind sole, with the objective of inducing external rotation of the foot (toe‐out) during the loading response phase of gait stance. For the fore sole, a variable stiffness sole was adapted, where a laterally stiffer sole design was developed to induce ankle eversion and lateral shift of foot centre of pressure, predominantly active during the second half of the stance phase.

The RVS shoe soles presented in this study were 3D‐printed using a geometric structure to allow stiffness variability as well as rotational functionality (Figure 1). The sole was designed in Rhinoceros 3D (version: 8, Robert McNeel & Associates, USA) and contained a neutral midsole base and a functional outsole, with a gyroid‐shaped infill structure [30]. The sole model was sliced in UltiMaker Cura (version: 5.4.0 UltiMaker B.V., Netherlands), and was then introduced into a fused filament 3D‐printer (Artillery 3D, Hong Kong) to fabricate the shoe soles using thermoplastic polyurethane. The outsole was divided into a hind‐, fore‐, and toe‐region. Specifically, a biomechanical rotation module with gyroid infill density of 30% was incorporated into the hind‐sole, with obliquely aligned filaments supporting a hollow core. These filaments were engineered to tilt under compressive forces, thereby inducing rotation of the wearer's foot under loading. The toe‐ and fore‐soles were fabricated using a variable stiffness design with a medial‐to‐lateral infill ratio of 5%‐to‐30%, separated along the mediolateral mid‐line of the outsole. The fore‐sole was designed to induce lateral shifting of the foot's COP during the mid‐ and terminal‐stance phases of gait. Following fabrication, the shoe sole was bonded to the shoe upper, with a neutral insole inserted to finalise the process. To ensure adequate fitting, appropriate shoe sizes were prepared for each subject.

### 2.3 Setup and Subject Preparation

The subjects abstained from any exhaustive exercise for a period of 48 h prior to data collection. Wearing athletic attire, subjects were each allowed approximately 2 min to familiarise themselves with each shoe. Eighteen reflective markers (14 mm) were then attached bilaterally to the subjects' shoulders, iliac crest, anterior superior iliac spine, posterior superior iliac spine, greater trochanter, lateral and medial femoral epicondyles, and lateral and medial tibial malleoli. Eight additional markers were affixed to the RVS and control shoes, positioned at the posterior calcaneus, the head of the first metatarsal, the head of the fifth metatarsal and the base of the second toe. Marker trajectory and GRF data were collected using a 20‐camera optical motion capture system (Vicon Motion Analysis Inc., UK; 200 Hz) and three 3D force plates (Kistler Holding AG, Switzerland; 2000 Hz).

### 2.4 Gait Measurements and Retraining

Gait measurements took the form of baseline calibration trials, followed by treadmill FPA gait retraining, and overground walking tests with the control or RVS shoes. Baseline tests involved the acquisition of three valid (clean foot‐strikes on the force plates) overground level walking trials at each subject's self‐selected velocity. The data from these three baseline trials were processed (see *Data processing*) to calculate gait velocity and natural FPA, which were used for setting up the gait retraining protocol for each subject.

**FIGURE 2:**
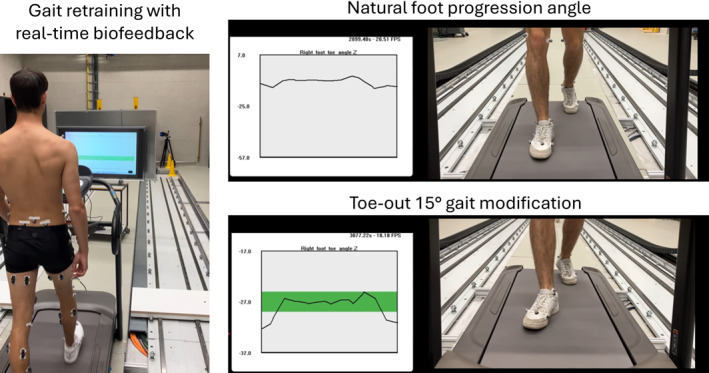
Gait retraining on the treadmill using a real‐time visual biofeedback system, where the target FPA was presented as a green band on the monitor for the subject to reproduce during gait.

Three gait retraining sessions (toe‐out‐5°, toe‐out‐10° and toe‐out‐15° [11, 14, 26]) were performed on a treadmill using the control shoe, at each subject's baseline gait velocity. A monitor was positioned in front of the treadmill to provide real‐time visual feedback (Figure 2), to guide subjects' gait patterns towards the targeted toe‐out angles (on top of their natural FPAs). Here, marker trajectory data were streamed in real time from the VICON Nexus system to Visual3D (HAS‐Motion, Ontario, Canada), where they were processed to calculate the FPA, presented as feedback curves on the monitor, together with a tolerance range of ± 2° from the target toe‐out angles. Subjects were instructed to adjust their foot position as well as possible to maintain their toe‐out angle within the tolerance range for each step during the 5‐min gait retraining session.

Three valid trials of overground level walking were then collected, where subjects were asked to replicate the trained gait pattern, while marker trajectory and GRF data were recorded. The order of the tests with different toe‐out angles (5°, 10°, 15°) was randomised, separated by a 5‐min wash‐out period. Subjects also performed three level walking trails wearing the RVS shoes, with the only instructions to walk compliantly with the shoes. After baseline testing, the order of the gait retraining and the RVS shoe tests was randomised across participants.

### 2.5 Data Analysis

The raw motion and force data were subjected to low‐pass filtering (Butterworth, fourth order, bidirectional) with cut‐off frequencies of 20 and 200 Hz using the Visual3D software suite. Calibration trials were utilised to determine joint centres and axes of rotation. The ankle joint centre was identified as the midpoint of the two malleoli markers, the knee joint centre as the midpoint of the two femoral epicondylar markers, and the hip joint centre using a geometric approach [31]. The motion trials were processed to compute key spatiotemporal parameters of gait, and lower‐limb joint kinematics and kinetics (Table 1). For reporting, joint angles were defined as the rotation of a given segment relative to its nearest proximal segment, following a Cardan sequence (*X*‐axis: medial → lateral, *Y*‐axis: posterior → anterior, and *Z*‐axis: distal → proximal). The polarity of rotation followed the right‐hand rule, with knee extension, adduction and internal rotation defined as positive. A gait cycle was defined between successive heel strikes of the same foot, identified using the GRF (threshold 10 N). The stance phase was defined as the period from heel‐strike to toe‐off accordingly. For each testing condition, the final results were averaged across the three valid gait trials and time‐normalised to each gait cycle, where appropriate. Joint moments and GRFs were normalised to height (m) × mass (kg) and weight (N), respectively.

**TABLE 1:** Definitions of gait and biomechanical variables of interest.

	Definitions
Gait spatiotemporal variables	
Step length (cm)	Distance from heel strike of the contralateral foot to heel strike of the ipsilateral foot along the line of progression
Step width (cm)	Mediolateral distance from the position of the ipsilateral foot's end at its heel strike to the position of the contralateral foot's end at the next heel strike
Stance phase (%)	Time from heel‐strike to toe‐off for the ipsilateral limb as a percentage of the full gait cycle
Gait velocity (m/s)	Average velocity of the body centre of mass throughout the gait cycle
Kinematic variables	
Trunk lean angle (°)	Trunk angle in the frontal plane, determined as the line from the mid‐point of the pelvis and mid‐point of the acromion markers with respect to the vertical. A positive value indicates a lean towards the side of the ipsilateral limb
Hip‐knee‐ankle angle (°)	Angle formed by the hip‐knee and knee‐ankle vectors projected in the frontal plane. A positive value indicates knee varus
Knee‐COP offset (cm)	Mediolateral distance between the vertical projection of the knee joint centre and the foot COP. A positive value indicates lateral offset of the knee
Ankle in/eversion angle (°)	Ankle angle in the frontal plane. A positive value indicates ankle inversion
FPA (°)	Angle between the vector connecting the heel and second toe markers and the line of walking progression. The baseline FPA was calculated as the average FPA during the mid‐stance phase of gait (time phase between the first and second peak GRFs)
Toe‐out angle (°)	External rotation angle of the foot in addition to each subject's baseline FPA
Kinetic variables	
First and second peak KAMs	Cross product of the frontal‐plane GRF and its lever arm relative to the knee joint centre (reported in the tibial reference frame), with its first and second peaks identified as the maxima occurring during the first and second halves of the stance phase
KAAI	Positive integral of the KAM over the stance phase of gait
Frontal‐plane GRF (/BW)	Magnitude of GRF in the frontal plane
GRF‐knee lever arm (cm)	Perpendicular distance from the knee joint centre to the GRF vector in the frontal plane
GRF angle (°)	Angle between the front‐plane GRF vector and the vertical coordinate axis. A positive value indicates medially tilting of the GRF vector
Mediolateral GRF (/BW)	Magnitude of mediolateral branch of the GRF. A positive value indicates medial GRF

### 2.6 Statistical Analysis

Statistical analysis was conducted using SPSS (version 28.0; SPSS Inc., USA). The significance level (*α*) was set a priori at 0.05. The results were presented as mean ± standard deviation (SD). One‐way RM‐ANOVA was conducted to examine differences in the variables across RVS shoes and different toe‐out conditions, with *Bonferroni*‐adjusted post hoc tests applied where appropriate. Effect sizes (ESs) were reported as partial eta‐squared measure (*η* ^2^), with effects classified as: 0.01—small, 0.06—medium, and 0.14—large.

Pearson correlation analyses were conducted to identify the associations between the change in the second peak KAM induced by the RVS shoes and variations in different joint kinematic and kinetic parameters, including FPA, knee‐COP offset, ankle in/eversion angle, hip‐knee‐ankle angle, trunk lean angle, GRF vector angle, and mediolateral GRF, compared to baseline gait. Multiple linear regressions (MLRs) were then conducted with the change in second peak KAM as the dependent variable, and the aforementioned kinematic and kinetic parameters set as the predictor variables. Parameters were processed using *Z*‐scores before being entered into the MLR model, in order to standardise the coefficients. To identify the optimal combination of predictors explaining the variation, a stepwise MLR approach was employed, using the *R* ^2^ increase as the criteria to determine the order of parameter entry.

## 3 Results

### 3.1 Gait Spatiotemporal Outcomes

Subjects exhibited very similar spatiotemporal gait patterns while walking overground with different toe‐out angles or RVS shoes. No significant differences were found in any spatiotemporal parameter (Table 2).

**TABLE 2:** Gait spatiotemporal variables when walking with natural foot progression angle compared to after retraining with different toe‐out angles and the RVS shoes.

Gait parameters	Natural FPA	Toe‐out‐5°	Toe‐out‐10°	Toe‐out‐15°	RVS shoe	*F*	*p*	ES
Step length (cm)	72.0 ± 8.9	71.5 ± 8.4	71.5 ± 8.8	71.4 ± 9.1	71.6 ± 7.8	0.193	0.941	0.010
Step width (cm)	10.9 ± 2.0	10.5 ± 2.2	11.0 ± 2.6	10.5 ± 2.6	10.9 ± 2.2	0.570	0.685	0.028
Stride length (cm)	144.5 ± 17.1	143.0 ± 16.0	142.6 ± 17.7	142.9 ± 17.7	143.0 ± 16.5	0.584	0.605	0.028
Stance phase (%)	62.2 ± 1.6	62.0 ± 1.4	62.1 ± 1.3	62.2 ± 1.5	62.4 ± 1.4	0.704	0.591	0.034
Gait velocity (m/s)	1.31 ± 0.22	1.29 ± 0.21	1.28 ± 0.21	1.29 ± 0.21	1.30 ± 0.22	0.701	0.594	0.034

Abbreviations: ES, effect size; *F*, *F*‐value; *p*, level of significance.

### 3.2 Knee Adduction Moment Outcomes

**FIGURE 3:**
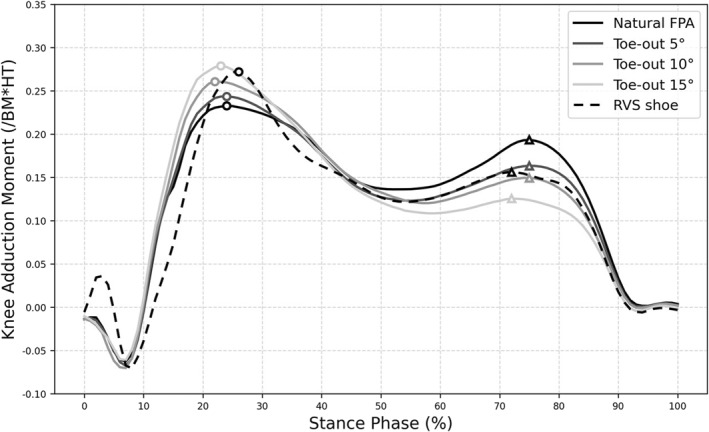
Knee adduction moment patterns during the stance phase of natural gait versus gait after retraining with different toe‐out angles, as well as wearing the RVS shoes. Circle and triangle symbols denote first and second peaks, respectively.

**FIGURE 4:**
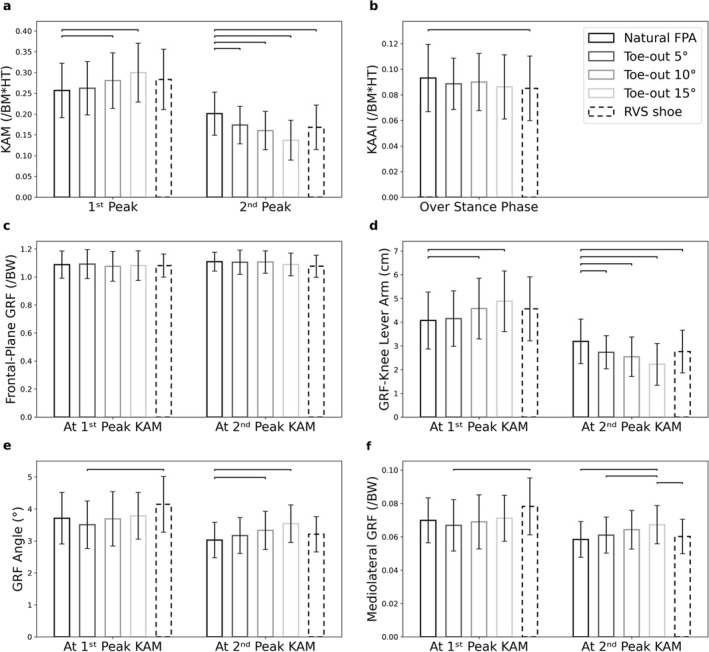
Comparisons of KAM and GRF variables between baseline gait, different FPAs, and RVS shoe. Horizontal lines denote significant pairwise differences. (a) First and second peak KAM; (b) KAAI; (c) frontal‐plane GRF; (d) GRF‐knee lever arm; (e) GRF vector angle; and (f) mediolateral component of the GRF.

A double‐bump pattern of KAM was observed during the stance phase of gait for all conditions (Figure 3). While the first peak KAM gradually increased with greater FPA, a progressive decrease in the second peak could be observed. The effect produced by the RVS shoes on the KAM reduction was analogous to toe‐out‐10°/15° gait retraining for the first peak, and between toe‐out‐5°/10° for the second peak. ANOVA revealed significant increases of up to 16.7% in the first peak KAM compared to baseline gait (Figure 4a). For the second peak KAM, significant reductions were found compared to baseline gait: RVS shoes (16.3%, *p* < 0.001), toe‐out‐5° (13.6%, *p* = 0.002), toe‐out‐10° (20.2%, *p* < 0.001), and toe‐out‐15° (31.7%, *p* < 0.001). Furthermore, significant differences were found in the KAAI (*p* < 0.001; Figure 4b), with RVS shoes showing a reduction of 8.6% (*p* < 0.001) compared to baseline.

### 3.3 Ground Reaction Force Outcomes

No significant differences were observed in the frontal‐plane GRFs at the instant of the first (*p* = 0.783) or the second peak KAM (*p* = 0.079) across the different walking conditions (Figure 4c). However, significant differences were observed in the length of the GRF‐knee lever arm at both the first (*p* < 0.001) and second peak KAMs (*p* < 0.001) (Figure 4d). At the first peak KAM, toe‐out‐10° and toe‐out‐15° significantly increased the lever arm by 9.8% (*p* = 0.010) and 19.9% (*p* < 0.001). At the second peak KAM, toe‐out‐5°, toe‐out‐10° and toe‐out‐15°, as well as RVS shoes significantly reduced the GRF‐knee lever arm by 14.4% (*p* = 0.002), 20.4% (*p* < 0.001), 30.4% (*p* < 0.001) and 13.5% (*p* = 0.002), respectively. In addition, ANOVA revealed significant differences in GRF vector angle at the first peak KAM, which was significantly larger in RVS shoes compared to toe‐out‐5° (*p* = 0.006, Figure 4e). At the second peak KAM, toe‐out‐10° (*p* = 0.050) and toe‐out‐15° (*p* < 0.001) exhibited significantly larger GRF vector angles than baseline gait. Finally, differences were found in the mediolateral GRF, where subjects exhibited significantly larger medial GRF at the first peak KAM while walking with RVS shoes compared to toe‐out‐5° (*p* = 0.017, Figure 4f). Similarly, subjects exhibited significantly larger medial GRFs at the second peak KAM with toe‐out‐5° (*p* = 0.024), toe‐out‐15° (*p* = 0.006) and the RVS shoes (*p* = 0.021) compared to baseline gait.

### 3.4 Kinematic Outcomes

**FIGURE 5:**
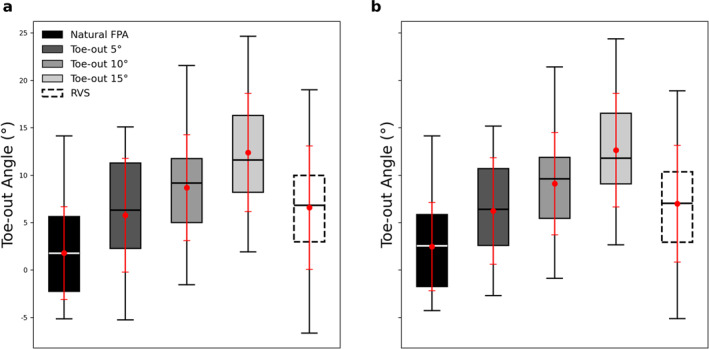
Box plot of average foot progression angles at the time point of the first (a) and second (b) peak KAM during overground level walking, with subjects instructed to replicate the toe‐out angle from gait retraining. Subjects were only instructed to walk compliantly with the RVS shoes. Whisker plots show median, interquartile ranges and maximum and minimum values, while the red labels show the mean and standard deviation of each condition.

ANOVA revealed significant differences in the FPA at the first (*p* < 0.001) and the second peak KAMs (*p* < 0.001), with all conditions exhibiting significantly greater toe‐out angles than baseline gait (*p* < 0.001) (Figure 5). Furthermore, subjects showed significantly different lower‐limb joint kinematics at the first and the second peak KAMs (Table 3).

**TABLE 3:** Kinematic variables during overground walking after retraining with different toe‐out angles, as well as gait measurements with the RVS shoes.

Kinematic parameters	Natural FPA	Toe‐out‐5°	Toe‐out‐10°	Toe‐out‐15°	RVS shoe	RM‐ANOVA			*p* of post hoc tests (vs. natural FPA)
						*F*	*p*	ES	
At first peak KAM									
Knee‐COP offset (cm)	1.0 ± 1.0	1.2 ± 1.0	1.4 ± 1.1	1.6 ± 1.1	1.0 ± 1.1	10.9	**< 0.001**	0.353	Toe‐out‐10°: 0.003
									Toe‐out‐15°: < 0.001
Ankle in/eversion angle (°)	−5.9 ± 2.2	−6.1 ± 2.0	−6.2 ± 2.0	−6.4 ± 2.4	−9.4 ± 2.5	20.3	**< 0.001**	0.504	RVS shoe: < 0.001
Hip‐knee‐ankle angle (°)	0.9 ± 2.4	0.1 ± 2.7	−0.6 ± 2.6	−1.3 ± 2.9	−0.2 ± 3.0	25.1	**< 0.001**	0.557	Toe‐out‐10°: < 0.001
									Toe‐out‐15°: < 0.001
									RVS shoe: 0.010
Trunk lean angle (°)	−0.5 ± 7.1	0.4 ± 8.4	1.3 ± 8.0	1.4 ± 8.6	−1.2 ± 8.7	2.90	0.104	0.127	
At second peak KAM									
Knee‐COP offset (cm)	0.6 ± 0.9	0.1 ± 0.8	−0.2 ± 1.0	−0.8 ± 1.0	0.0 ± 1.0	27.1	**< 0.001**	0.576	Toe‐out‐5°: 0.002
									Toe‐out‐10°: < 0.001
									Toe‐out‐15°: < 0.001
									RVS shoe: < 0.001
Ankle in/eversion angle (°)	−6.4 ± 2.2	−4.8 ± 2.5	−4.7 ± 2.1	−3.4 ± 2.5	−7.2 ± 2.4	23.6	**< 0.001**	0.542	Toe‐out‐5°: 0.009
									Toe‐out‐10°: 0.029
									Toe‐out‐15°: < 0.001
Hip‐knee‐ankle angle (°)	−0.5 ± 2.3	−1.0 ± 2.2	−1.1 ± 2.3	−1.5 ± 2.2	−1.0 ± 2.2	7.70	**< 0.001**	0.278	Toe‐out‐10°: 0.017
									Toe‐out‐15°: 0.011
Trunk lean angle (°)	1.7 ± 7.4	0.4 ± 5.9	0.4 ± 4.5	1.0 ± 4.9	−0.7 ± 6.8	0.67	0.570	0.032	

Abbreviations: ES, effect size; *F*, *F*‐value; *p*, level of significance. Bold values denote significant differences between the testing conditions.

### 3.5 Correlation Outcomes

**FIGURE 6:**
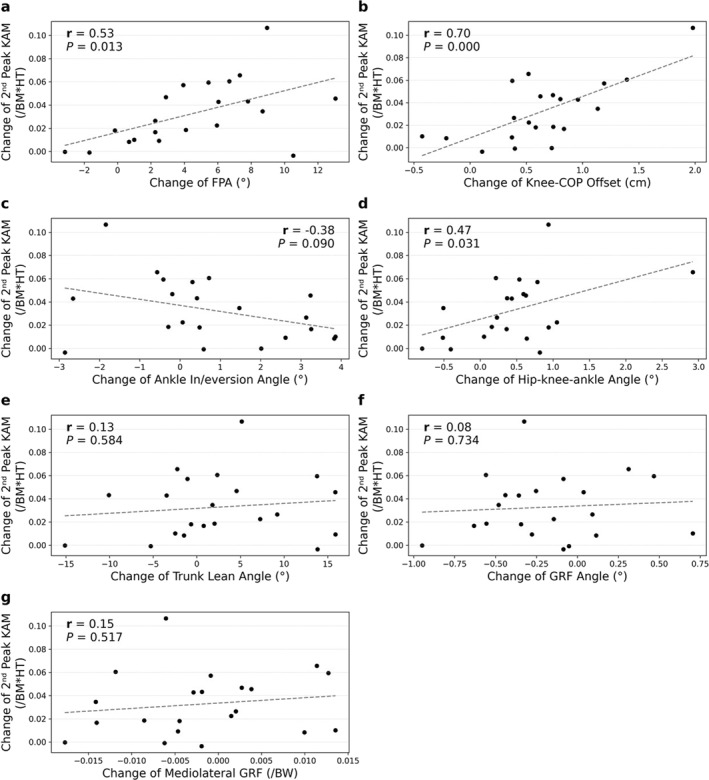
Correlations between the change of the second peak KAM (RVS shoe vs. natural gait) and changes of (a) FPA; (b) knee‐COP offset; (c) ankle in/eversion angle; (d) hip–knee–ankle angle; (e) trunk lean angle; (f) GRF vector angle; and (g) mediolateral component of the GRF.

Strong correlations were observed with the RVS shoes between the change of second peak KAM and the knee‐COP offset, while moderate correlations were seen for FPA and hip‐knee‐ankle angle, and moderate negative correlations were found for ankle in/eversion angle (Figure 6). All correlations for the other variables were negligible. MLR analysis further revealed that knee‐COP offset and mediolateral GRF were the two main predictors for the change of second peak KAM in the RVS shoes, together explaining over 95% of the variation in the dependent variable (Table 4).

**TABLE 4:** Multiple linear regression analysis between change of second peak KAM (RVS shoe vs. natural gait) and changes of biomechanical variables.

Dependent variable	Predictors	*R* ^2^	Adjusted *R* ^2^	Coefficients (95% CI)	*F*	*t*	*p*
Change of second peak KAM	Knee‐COP offset	0.495	0.468	0.0368 (0.019, 0.055)	18.6	4.32	< 0.001
	Knee‐COP offset	0.962	0.957	0.0320 (0.029, 0.035)	225.0	21.0	< 0.001
	Mediolateral GRF			0.0225 (0.019, 0.026)		14.8	
	Knee‐COP offset	0.965	0.959	0.0330 (0.029, 0.037)	156.7	19.6	< 0.001
	Mediolateral GRF			0.0230 (0.020, 0.026)		15.0	
	Ankle in/eversion			0.0018 (−0.001, 0.005)		1.32	

*Note:* The order of parameter entry was determined based on the *R* ^2^ increase within a stepwise MLR approach.

## 4 Discussion

In the present study, a prototype shoe with a 3D‐printed sole has been developed with the objective to replicate the orthotic functionality of toe‐out gait modifications. The findings revealed that wearing the so‐called RVS shoes elicited substantial reductions in KAM variables, which were comparable to, or even exceeded, those achieved via toe‐out gait retraining at various targeted FPAs.

This study implemented a gait retraining approach using real‐time visual biofeedback [22], which trained the subjects to walk with three different FPAs (toe‐out‐5°/10°/15°), representing the most commonly investigated targets [11, 13, 14]. In this study, subjects underwent gait retraining at each of the targeted FPAs, and demonstrated average toe‐out modifications of 4.0°, 6.9° and 10.6° during subsequent overground walking trials. These results are consistent with earlier findings, such as average FPA modifications of 6.7° and 8.9° following training at toe‐out‐10° [16] and toe‐out‐15° [17]. These results suggest that replicating larger toe‐out modifications may be more challenging than smaller FPAs in the context of no supervision, potentially compromising their biomechanical efficacy. In this study, gait retraining achieved average reductions in the second peak KAM ranging from 13.6% to 31.7%, which is consistent with previous studies [13, 14, 32]. These effects were clearly achieved through shortening the GRF's lever arm to the knee centre at the second peak KAM [33]. These findings confirm that the employed gait retraining protocol induced substantial modifications in subjects' FPAs and KAM magnitudes, thus providing a robust basis for comparison with the biomechanical effects of the RVS shoes.

Previous studies have reported that reductions in the second peak KAM correspond to reductions in the second peak medial tibiofemoral contact force, whereas reductions in the first peak KAM may not consistently show such an association [34]. Therefore, the RVS shoe sole was specifically designed to induce a toe‐out gait modification to target reductions in the second peak KAM. Here, the RVS shoe achieved a significant reduction of 16.3% during overground level walking, positioning its biomechanical effect between gait retraining at toe‐out‐5° and toe‐out‐10°. A notable advantage of the RVS shoe, however, lies in its ability to passively induce the desired toe‐out effect during walking, thereby eliminating the need for any active training intervention. Therefore, while the long‐term effects of the RVS shoe remain to be investigated, it is plausible that its biomechanical efficacy does not diminish over time, contrary to that observed with active gait retraining. The RVS shoe also demonstrates superior effects over the conventional LWI currently used in clinical practice, which has been reported to reduce the second peak KAM by approximately 3%–11% [35, 36, 37, 38, 39]. Moreover, it is likely that a flat soled shoe design remains comfortable, and is therefore less affected by the same adherence problems associated with LWIs [39, 40].

In this study, correlation analysis was conducted between the change of second peak KAM induced by the RVS shoes and joint kinematic/kinetic variables either independently or as a combination, compared to baseline gait. Modifications to the FPA and ankle in/eversion angles were targeted by the RVS shoes during gait stance. However, only moderate correlations were observed, indicating only mild influence of ankle kinematic alterations on the variation of the second peak KAM. Additionally, moderate correlations were observed for the hip‐knee‐ankle angle. This finding was somewhat surprising, as only an insignificant reduction in knee varus angle was observed with the RVS shoes. It seems that the combination of sole stiffness and geometry, together with a modified curved COP progression and passively changing FPA, all result in complex interactions. Further investigation into understanding these effects is clearly warranted.

Despite almost no change in the knee varus angle, a significant reduction in knee‐COP offset at the second peak KAM was observed with the RVS shoes, indicating a lateral shift of the foot COP with respect to the knee joint centre [41]. This is of importance, as it suggests that the GRF‐knee lever arm is shortened, which may provide a rationale for the strong correlation observed between variations in the second peak KAM and the knee‐COP offset. Furthermore, the outcomes of the MLR analysis indicate a strengthening of this correlation when in combination with the mediolateral GRF vector, even though this parameter alone exhibited negligible correlations. Since these two parameters dominated the location and direction of the frontal‐plane GRF vector, it is plausible that they act synergistically on the second peak KAM. Nevertheless, this important finding merits further investigation towards facilitating the prediction of KAM variations for biomechanical interventions.

The KAAI has been reported to be independently associated with cartilage volume loss in a 12‐month follow‐up study [7], warranting attention as a biomechanical risk factor for KOA. The RVS shoe was found to significantly reduce the KAAI by 8.6%, while none of the three toe‐out gait retraining strategies could demonstrate statistically meaningful reductions in this parameter. It appears that the onset of the KAM with the RVS shoes was somewhat delayed compared to the other conditions (Figure 3), providing a rationale for the significant reduction in the KAAI observed. This delayed KAM curve is likely attributable to a softer heel region of the shoe sole associated with the rotary effect of the rotation module. Similar effects plausibly result in the 7.7% [36, 37, 42] and 4.5% [43] KAAI reductions reported in the LWI and rocker‐sole shoe. On the other hand, the AposTherapy shoe was reported to achieve KAAI reductions of up to 14.0% [44]. Importantly, however, the AposTherapy shoe should be interpreted as a complex intervention rather than a purely passive orthotic device, as it integrates a personalised biomechanical footwear platform with a structured programme of progressive adaptation and neuromotor training. In addition, their study utilised the same AposTherapy shoe without the attached element as their control condition, which is not representative of a control condition with a uniform neutral shoe or the subject's own shoes. Consequently, these findings support the biomechanical efficacy of the RVS shoe as a passive orthosis in modulating the KAAI during functional activities.

The present study demonstrated unfavourable increases in the first peak KAM for all FPA gait retraining conditions, with statistical significance observed in the toe‐out‐10° and toe‐out‐15° modifications, as similarly reported in previous studies [13, 14, 32]. Meanwhile, the increased first peak KAM likely compromised the effect of the reduced second peak KAM on diminishing the aggregate KAAI, thereby explaining the non‐significant KAAI variations between the different gait retraining strategies and baseline gait. Similarly, an increase in the first peak KAM was observed during gait with the RVS shoes, although this increase was not statistically significant. In contrast to gait retraining, this increase is unlikely to be associated with the knee‐COP offset (since this remains unchanged), but rather with the greater medial GRF vector.

The present study was not without limitations. Firstly, as the first study using 3D‐printed rotation soles, gait measurements were conducted on healthy subjects to reduce ethical concerns and facilitate quicker design modifications. Subsequent studies should include KOA patients to further validate the real‐world efficacy of the RVS shoes. Secondly, this study only investigated subjects' immediate biomechanical response to the RVS shoes in the frontal plane. Future studies should explore the comprehensive 3D kinematics and kinetics of the lower limbs, as well as the long‐term effects of the RVS shoes. Finally, only a single design RVS shoe sole was developed and tested, whereas personalised designs of the sole's geometric and stiffness features could likely provide more optimised biomechanical efficacy.

## 5 Conclusions

The present study developed and evaluated RVS shoes featuring a 3D‐printed rotational hind‐sole and a variable stiffness fore‐sole, with the aim to reduce KAM variables during ambulation. In comparison to toe‐out gait retraining, the RVS shoe sole demonstrated comparable reductions in second peak KAM and greater reductions in KAAI in healthy individuals, thereby underscoring its potential as a viable conservative intervention. Future research should explore KOA patient‐specific responses to the RVS shoes to enable optimisation of sole design.

## Author Contributions

**Ziang Jiang:** formal analysis, writing – original draft preparation. **Paciane Bo Studer:** project administration. **Matthias Zäh:** investigation, methodology. **Christian Kryenbühl:** resources. **Stephan Reichenbach:** conceptualization, validation. **William R. Taylor:** conceptualization, funding acquisition, writing – review and editing. **Qiang Zhang:** data curation, formal analysis, supervision, visualization, writing – original draft preparation.

## Funding

This study was partially funded by the Innosuisse (Grant 104.374 IP‐LS).

## Ethics Statement

This study was conducted in accordance with the Declaration of Helsinki. This study was approved by the ETH Zurich Ethics Commission (EK‐2024‐N‐146) before subject recruitment. All subjects provided written informed consent prior to participation.

## Consent

The authors have nothing to report.

## Conflicts of Interest

The authors declare no conflicts of interest.

## Data Availability

The datasets used and/or analysed during the current study are available from the corresponding author on reasonable request.
